# Analysis of serum calcium change trajectories and prognostic factors in patients with acute type A aortic dissection

**DOI:** 10.1186/s12893-023-02249-3

**Published:** 2023-11-27

**Authors:** Jian-Long Lin, Sai-Lan Li, Yan-Chun Peng, Liang-Wan Chen, Yan-Juan Lin

**Affiliations:** 1 Department of Cardiovascular Surgery, Fujian Medical University Union Hospital, No.29 Xinquan Road, Fuzhou, Fujian 350001 China; 2 Department of Nursing, Fujian Medical University Union Hospital, No.29 Xinquan Road, Fuzhou, Fujian 350001 China

**Keywords:** Type A acute aortic dissection, Serum calcium, Poor prognosis, Risk factors

## Abstract

**Objectives:**

This study aimed to analyze the correlation between serum calcium changes and short-term prognosis of patients with acute type A aortic dissection.

**Methods:**

Patients who underwent acute type A aortic dissection surgery at Fujian Heart Medical Center between June 2019 and June 2021 were retrospectively analyzed.

**Results:**

A total of 383 patients were enrolled. According to the changing track of serum calcium in patients after acute type A aortic dissection, three potential category tracks were determined: high-level (*n* = 85), medium-level (*n* = 259), and continuous low-level groups (*n* = 39). Using the medium-level group as the control, regression analysis showed that poor prognosis risk was increased in the group with continuous low serum calcium (odds ratio = 2.454, *P* < 0.05) and in the group with continuous low serum calcium > 48 h (odds ratio = 3.595, *P* < 0.05). Age (odds ratio = 1.063, *P* < 0.001), body mass index (odds ratio = 1.138, *P* < 0.05), hypertension (odds ratio = 3.697, *P* < 0.05), and the highest lactic acid within 72 h after surgery(odds ratio = 1.093, *P* < 0.05) were independent risk factors for poor prognosis after aortic dissection.

**Conclusion:**

Continuous low serum calcium was an independent predictor of poor prognosis in patients with acute type A aortic dissection.

**Supplementary Information:**

The online version contains supplementary material available at 10.1186/s12893-023-02249-3.

## Key points

Key Question

What is the relationship between serum calcium and poor patient prognosis in acute type A aortic dissection (AAAD).

Key finding(s)

Of three categories, continuous low calcium for more than 48 h is related to poor prognosis of patients with AAAD.

Take-home message

Clinicians should determine causes of low calcium and prevent fluctuations postoperatively, thereby decreasing incidence of poor prognosis.

**Supplementary Information:**

The online version contains supplementary material available at 10.1186/s12893-023-02249-3.

## Introduction

Acute aortic dissection (AAD) is an acute and critical cardiovascular disease (CVD). Dissection involving the arterial root, ascending aorta, or aortic arch is classified as Stanford type A or B. According to survey results in China from 2015 to 2016 [1], the incidence of AAD is 2.78/100000, and the average age is lower than that reported in Western countries. Approximately 2/3 of AAD cases are type A aortic dissections (AAADs) [2] which is characterized by rapid disease development and high mortality. Rapid diagnosis and emergency surgical treatment can save patient lives [3]. Although significant progress has been made in perioperative nursing management and surgical techniques in AAAD, complications such as death, gastrointestinal bleeding, and paraplegia continue to decrease the quality of life and place significant burdens on families and society [4].

Recent research has focused on the predictive value of serum calcium levels on clinical outcomes. Early changes in serum calcium are an independent risk factor for mortality in patients with acute myocardial infarction and heart failure [5, 6]. Wang et al. [7] found that low calcium ion concentration is an independent predictor of all-cause mortality and may be a potential biomarker for the prognosis of patients with acute kidney injury (AKI) by determining the severity of renal function impairment. Appel et al. [8] found that abnormal serum calcium was related to the risk of progression and poor prognosis of patients with acute ischemic stroke. Polderman et al. [9] showed that after cardiac surgery, 88% of patients had a significant decrease in one or more electrolytes. Serum calcium levels are closely related to vascular smooth muscle contraction, muscle movement, nerve conduction, and blood coagulation pathways. Serum calcium is vital for the stability of the cardiovascular system and blood–brain barrier. Considering the influence of serum calcium on the prognoses of patients who are categorized as acute and severe, we hypothesized that serum calcium may be associated with the outcome and prognosis of patients with AAAD.

Currently, most studies only focus on the serum calcium level of patients with AAAD at admission. To our knowledge, no research has focused on the regularity of the change in serum calcium levels and on whether different tracks of serum calcium change were related to clinical outcomes.

## Patients and methods

### Ethical statement

This study was reviewed by the Fujian Medical University Hospital Ethics Committee (No.2020KY082) and complied with the Declaration of Helsinki.

### Study design

This was an observational retrospective study. Patients with AAAD at Fujian Heart Medical Center were recruited between June 2019 and June 2021. Inclusion criteria were: (1) computed tomography angiography and magnetic resonance imaging were used to diagnose AAAD [10]; (2) time from onset to admission was < 24 h; (3) postoperative patients with aortic dissection; and (4) age ≥ 18 years. The exclusion criteria were as follows: (1) thyroid disease or end-stage renal disease (estimated glomerular filtration rate [eGFR] < 15 mL/min/1.73), blood system diseases, or severe infections and other malignancies; (2) pregnancy; (3) long-term use of drugs that may affect blood results; and (4) incomplete medical records.

### Data collection

Data collected included patient, preoperative, operative, and postoperative variables of interest. Sociodemographic data, disease information, and laboratory examination data of patients were collected by reviewing the electronic case system. Sociodemographic data included age, sex, body mass index (BMI), smoking status, and drinking history. Disease information included hypertension, diabetes, pre-existing malperfusion in preoperative brain/chest/abdomen/pelvis CT scans, and left ventricular ejection fraction (LVEF). Laboratory examination data included admission hemoglobin, C-reactive protein, B-type natriuretic peptide, creatinine, serum albumin, serum potassium, serum sodium, serum chloride and serum calcium. Intraoperative data included surgical management (root replacement, additional CABG), cardiopulmonary bypass (CBP) and operative times, and the use of calcium infusions in the operating room. Postoperative data included the highest lactic acid values within 72 h after surgery, doses of calcium and vasoactive medications given during the perioperative periods were collected. For the calculation of the vascular active drug usage peak 72 h after surgery reports, the vasoactive inotropic score (VIS) [11] was used. Simultaneously, blood biochemical serum calcium values were collected 24, 48, and 72 h after surgery. If serum calcium values were repeated within 24 h, the interval was close to 24 h. The main outcome indicators of poor prognosis were postoperative death and major adverse cardiac and cerebrovascular events (MACCES) including new arrhythmias, cardiac insufficiency, acute pericardial tamponade, disturbance of consciousness, dyskinesia, intracerebral hemorrhage or hematoma, cerebral infarction, transient ischemic attack, and central nervous system infection. Secondary outcome measures included postoperative AKI, postoperative pulmonary complications (re-endotracheal intubation, tracheotomy, pleural effusion, pneumothorax and hemothorax, pulmonary infection, and respiratory failure), gastrointestinal bleeding, prolonged intensive care unit (ICU) mechanical ventilation, stay time, and postoperative hospital stay. Hypocalcemia was defined as a serum calcium level < 2.25 mmol/L. Prolonged mechanical ventilation (PMV) was defined as a duration of mechanical ventilation exceeding 48 h. Postoperative AKI was defined as an increase in serum creatinine (Scr) ≥ 0.3 mg/dl within 48 h or Scr ≥ 1.5 times the baseline level within seven days.

### Statistical analysis

To identify heterogeneity in the patterns of the subgroups among patients, the unconstrained latent category growth model (LCGM) was used to judge the trajectory category and category characteristics, increase the number of categories of the model individually, and compare the fitting indicators between models. A P value less than 0.05 indicates that the k-category model is superior to the k-1 category model. The measurement data with normal distribution are expressed as mean ± standard deviation. Continuous variables were analyzed using analysis of variance, and categorical variables were tested using the chi-square test. The measurement data of non-normal distribution were compared using the Kruskal Wallis test and are expressed as median (interquartile interval). The chi-square test was used for counting data which are expressed as percentages. To determine the risk factors, logistic regression analysis was used to explore the impact of social demography, disease-related data, and laboratory tests on poor prognosis. The correlation between adverse prognostic factors was evaluated by calculating the odds ratios (ORs) and 95% confidence intervals (CIs). Subgroup analysis was used to investigate the potential impact of heterogeneity on poor outcomes. Moreover, The area Under the Curve (AUC) of Receiver Operating Characteristic Curve (ROC) was used to evaluate the model differentiation, the Hosmer–Lemeshow goodness of fit test (H–L test) and calibration curve were used to evaluate the calibration degree of the model. R software (version 3.4.1) and Mplus8.3 were used for data analysis. The test level was set for both sides at α = 0.05, and *P* < 0.05 was considered statistically significant.

## Results

### Patient clinical characteristics

**Fig. 1:**
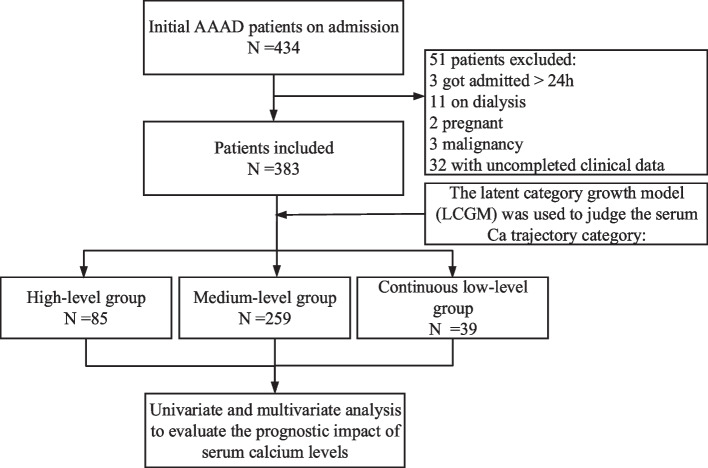
Study flow chart

Among 434 patients with AAAD, 51 patients were excluded because of the following reasons. 3 patients time from onset to admission > 24 h, 11 patient on dialysis, 2 patients with malignancy, 3 patients had comorbidity of cancer, and 32 patients with incomplete data. Ultimately, 383 patients were included (Fig. 1). The mean age was 50.01 ± 8.26 years (321 men; 62 women). The median interval between two adjacent serum calcium measurements was 1.1 (interquartile range [IQR] 0.6–2.2) days. The serum calcium levels of 20 patients were < 2.25 mmol/L at admission (T1), 24 h (T2), 48 h (T3), and 72 h (T4). Thirty patients had low calcium levels within 24 h, 15 within 24–48 h, and 22 within 48 h. For the group with hypocalcium, 289 cases of AAAD occurred within 24 h, 53 within 24–48 h, and 41 occurred after 48 h. The prognosis was poor in 80 cases, including 66 deaths, 48 renal failures and 11 patients needing renal repalcement therapy within 72 h(Table 3).

### Identification and determination of trajectory types of serum calcium changes

**Fig. 2:**
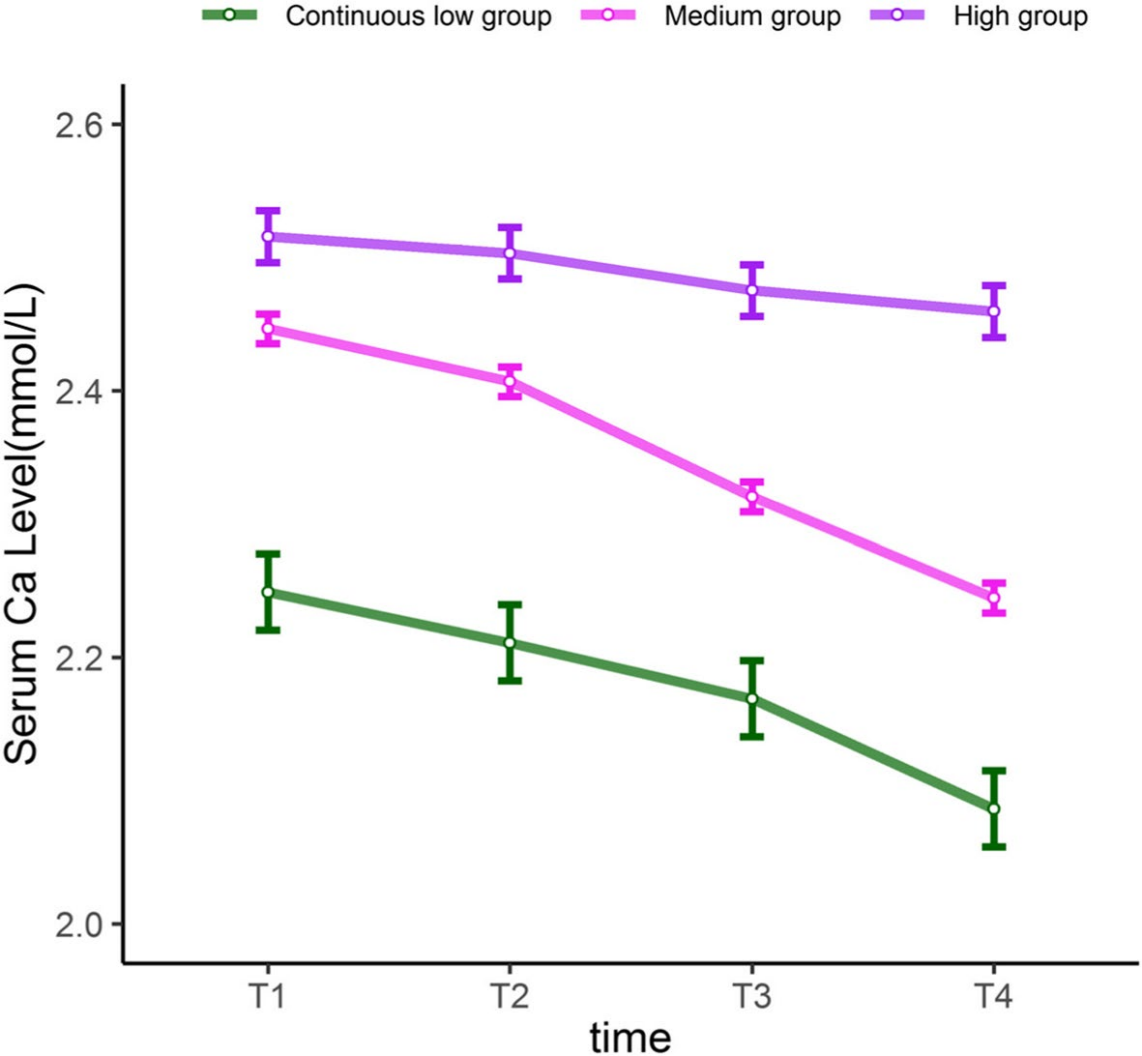
Latent category growth model trajectory for mean serum calcium in patients with type A acute aortic dissection

There was a significant correlation between the serum calcium levels of patients with AAAD four times, and the effect size was small to moderate (*r* = 0.264–0.565). This reflects the continuity of the serum calcium levels. From the overall average trend, the serum calcium levels showed a significant downward trend within 24–72 h after surgery (Fig. 2). The serum calcium levels at four different time points were used as observation indicators. The LCGM was used to set a free estimation of the time parameters, and 1–5 categories were extracted. When the potential category number was increased from 1 to 3, AIC, BIC, and aBIC decreased, and LRT and BLRT reached significant levels (*P* < 0.001). AIC, aBIC, and BIC values increased from four to five categories, and BLRT reached a significant level in five categories (*P* > 0.05). Finally, three categories of the LCGM were retained (Table 1).

**Table 1:** Fitting of latent category growth model of serum calcium in patients with type A acute aortic dissection

No. of profiles	AIC	BIC	a-BIC	Entropy	BLRT(*P*)	LMR(*P*)	N
1	-597.140	-573.452	-592.489	-	-	-	-
2	-847.780	-812.248	-840.803	0.751	< 0.001	< 0.001	269/114
3	-972.341	-924.964	-963.038	0.790	< 0.001	< 0.001	85/259/39
4	-973.176	-913.955	-961.548	0.605	0.624	0.605	81/40/197/65
5	-988.080	-917.015	-974.127	0.740	0.524	0.545	81/68/189/1/44

The LCGM can capture information about interindividual differences in the change of serum calcium levels over time and identify participants with similar serum calcium levels trajectories. Group l (purple) showed high serum calcium levels at admission (T1) (I = 2.515). However, the average change in the whole T1–T4 was not significant (S = -0.015, *P* = 0.121); therefore, this was considered a "high-level group". Although the initial value of group 2 (red) was moderate (I = 2.446), it showed a downward trend in the follow-up (S = -0.081, *P* < 0.001). Therefore, group 2 was considered a "continuous medium-level group". The serum calcium levels of patients in group 3 (green) were maintained at a low level. Therefore, group 3 was considered a "continuous low-level group" (I = 2.248, S = -0.030, *P* < 0.001) (Fig. 2).

### Comparison of clinical data

There were significant differences in BMI, LVEF, serum chloride, and lactic acid among the three groups (*P* < 0.05). There were no significant differences in age, sex, smoking and drinking history, diabetes, or pre-existing malperfusion (*P* > 0.05). There were no significant differences in the admission hemoglobin, C-reactive protein, B-type natriuretic peptide, creatinine, serum sodium, or serum potassium (*P* > 0.05). Furthermore, the serum chloride level and LVEF were lower in the low serum calcium group than those in the other groups, whereas BMI and lactic acid levels were higher. The baseline patient characteristics are shown in Table 2.

**Table 2:** Clinical data in three groups stratified by serum calcium trajectories

Variables	Total (*n* = 383)	High group (*n* = 85)	Medium group (*n* = 259)	Continuous low group (*n* = 39)	*P* -value
Age(years), mean (SD)	49.1 ± 8.0	48.7 ± 7.8	49.1 ± 7.9	49.4 ± 9.1	0.862
Female, n, (%)	62(16.2)	11(12.9)	44(17.0)	7(17.9)	0.647
BMI (kg/m^2^), mean (SD)	24.7 ± 3.4	24.6 ± 3.4	24.6 ± 3.3	26.1 ± 3.3	**0.033**
Smoking, n, (%)	184(48.0)	47(55.3)	122(66.3)	15(38.5)	0.190
Drinking, n, (%)	123(32.1)	23(27.1)	85(32.8)	18(46.2)	0.110
Medical history					
Diabetes mellitus, n, (%)	41(10.7)	12(14.1)	24(9.3)	5(12.8)	0.411
Hypertension, n, (%)	329(85.9)	73(81.2)	224(86.5)	36(92.3)	0.227
Prior resuscitation, n, (%)					
≥ 3 organs, n, (%)	9(2.3)	2(2.4)	4(1.5)	3(7.7)	0.061
Echocardiography results					
LVEF (%), mean (SD)	62.34 ± 7.75	62.88 ± 7.55	62.82 ± 7.34	57.96 ± 9.50	**0.001**
Laboratory results					
Hb(g/L), mean (SD)	125.58 ± 18.33	128.55 ± 20.25	125.48 ± 19.11	119.76 ± 17.75	0.875
CRP (mmol/L), median (IQR)	13.6(5.2, 32.0)	16.0(6.7, 28.8)	12.0(5.2, 34.2)	12.0(4.5, 36.0)	0.648^a^
Cr(mmol/L), median (IQR)	90(73, 132)	92(70, 134)	89(73, 132)	93(77, 131)	0.670^a^
BNP (mmol/L), median (IQR)	328(128, 934)	328(135, 858)	326(117, 986)	365(128, 920)	0.983^a^
Serum albumin(g/l), median (IQR)	43(38,49)	38(36,53)	43(39,51)	43(37,51)	0.958^a^
Serum potassium(mmol/l), mean (SD)	4.17 ± 0.56	4.06 ± 0.48	4.21 ± 0.59	4.13 ± 0.45	0.083
Serum sodium(mmol/l), mean (SD)	136.39 ± 5.87	136.28 ± 6.12	136.51 ± 6.00	135.82 ± 4.29	0.782
Serum chloride(mmol/l), mean (SD)	98.14 ± 7.23	98.82 ± 11.80	98.33 ± 5.20	95.43 ± 5.08	**0.040**
Surgical management					
Root replacement, n, (%)	14(3.7)	3(3.5)	8(3.1)	3(7.7)	0.395
Additional CABG, n, (%)	9(2.3)	3(3.5)	5(1.9)	1(2.6)	0.481^b^
Operation time(min), median (IQR)	305(265, 361)	300(265, 387)	305(270, 353)	319(262, 355)	0.955^a^
CPB time(min), median (IQR)	151(132, 189)	152(133, 203)	153(130, 188)	147(134, 185)	0.791^a^
Calcium infusion(g), median (IQR)	72(55, 100)	78(54, 104)	72(55, 97)	67(49, 127)	0.849^a^
Calcium infusion initiated in OR, n, (%)	48(12.5)	9(10.6)	31(12.0)	8(20.5)	0.268
Lactic acid_max_ (mmol/l), median (IQR)	3.7(2.3, 6.6)	4.1(2.7, 8.2)	3.5(2.0, 6.4)	4.5(3.2, 7.0)	**0.003**^a^
VIS_max_	9.4(2.9, 20.0)	8.6(3.0, 20.0)	9.0(2.8, 20.0)	13.3(4.4, 20.0)	0.344^a^

*BMI* body mass index, *LVEF,left* ventricular ejection fraction, *HB* hemoglobin, *CRP* C-reactive protein; Cr,creatinine, *BNP* B-type natriuretic peptide, *CABG* coronary artery bypass graft, *CBP* cardiopulmonary bypass, *OR* operating room, *VIS* vasoactive inotropic score

^a^Kruskal-Wallis H; ^b^Fisher's exact test

### Comparison of postoperative outcomes and complications

There was no significant difference in ICU hospitalization time and days among the three groups (*P* > 0.05). There were significant differences between severe cardiovascular and cerebrovascular events (7.1 vs. 6.6 vs. 17.9%), PMV (9.4 vs. 13.1 vs. 25.6%), AKI (41.2 vs. 44.0 vs. 64.1%), postoperative new arrhythmia (4.7 vs. 2.3 vs. 12.8%), and death (11.8 vs. 17.0 vs. 30.8%) (*P* < 0.05); 15 patients (17.6%) had poor prognosis in the high serum calcium level group, 49 patients (18.9%) in the medium-level group, and 16 patients (41.0) in the low-level group. The differences between the three groups were statistically significant (*P* < 0.05) (Table 3).

**Table 3:** Poor prognosis in three groups stratified by serum calcium trajectories

Outcomes	Total (*n* = 383)	High group (*n* = 85)	Medium group (*n* = 259)	Continuous low group (*n* = 39)	*P* -value
ICU stay time(day), median (IQR)	7(4, 11)	6(4, 12)	7(4, 11)	6(4, 12)	0.090^a^
Hospital stays(day), median (IQR)	22(17, 31)	21(16, 30)	23(17, 34)	23(16, 32)	0.077^a^
Death in hospital, n, (%)	66(17.2)	10(11.8)	44(17.0)	12(30.8)	**0.033**
In-hospital Complications					
MACCEs, n, (%)	32(8.4)	6 (7.1)	17(6.6)	9 (17.9)	**0.002**
Pulmonary complications, n, (%)	138 (36.0)	27(31.8)	94(36.3)	17(43.6)	0.439
Gastrointestinal bleeding, n, (%)	40 (10.5)	9(10.6)	25(9.7)	6 (15.4)	0.556
Acute kidney injury, n, (%)	174(45.4)	35(41.2)	114(44.0)	25(64.1)	**0.043**
Prolonged MV, n, (%)	52(13.6)	8(9.4)	34(13.1)	10(25.6)	**0.046**
Arrhythmia, n, (%)	15(4.4)	4(4.7)	6(2.3)	5(12.8)	**0.006**
Poor prognosis, n, (%)	80(20.9)	15(17.6)	49(18.9)	16(41.0)	**0.005**

^a^Kruskal-Wallis H

### Univariate and multivariate analysis

Variables with *P* < 0.2 were included in the multivariate regression analysis to screen for independent risk factors. This analysis showed that age (OR = 1.063, 95% CI = 1.029–1.098, *P* < 0.001), BMI (OR = 1.138, 95% CI = 1.047–1.237, *P* = 0.002), hypertension (OR = 3.697, 95% CI = 1.211–11.189, *P* = 0.021), and lactic acid level (OR = 1.093, 95% CI = 1.022–1.169, *P* = 0.010) were independent risk factors of poor prognosis. Compared with the medium-level group, the continuous low-level group (OR = 2.454, 95% CI = 1.074–5.604, *P* = 0.033) showed an increased risk of poor prognosis. Compared with continuous low calcium levels for < 24 h, low calcium levels for > 48 h (OR = 3.595, 95% CI = 1.597–8.093, *P* = 0.002) also increased the risk of poor prognosis (Table 4).

**Table 4:** Univariate/multivariate comparison of poor prognosis groups

Variable	Univariate model		Multivariate model	
	*OR* (95%CI)	*P* value	*OR* (95%CI)	*P* value
Age(years)	1.067 (1.036–1.099)	** < 0.001**	1.063 (1.029–1.098)	** < 0.001**
Female	0.893 (0.450–1.771)	0.746	-	-
BMI (kg/m^2^)	1.167 (1.081–1.261)	** < 0.001**	1.138 (1.047–1.237)	**0.002**
Smoking	1.037 (0.633–1.697)	0.887	-	-
Drinking	1.050 (0.623–1.769)	0.855	-	-
Medical history				
Diabetes mellitus	0.621 (0.252–1.532)	0.301	-	-
Hypertension	3.755 (1.314–10.732)	**0.014**	3.697 (1.222–11.189)	**0.021**
Prior malperfusion				
≥ 3 organs, n, (%)	1.929 (0.472–7.887)	0.361	-	-
Echocardiography results				
LVEF	0.976 (0.948–1.006)	0.117	0.985 (0.951–1.019)	0.380
Laboratory results				
Hb	0.994 (0.980–1.007)	0.351	-	-
CRP	0.998 (0.991–1.005)	0.609	-	-
Cr	1.001 (0.998–1.003)	0.757	-	-
BNP	1.000 (1.000–1.000)	0.626	-	-
Serum albumin	1.001 (0.979–1.023)	0.950	-	-
Serum potassium	0.854 (0.546–1.337)	0.491	-	-
Serum sodium	1.001 (0.964–1.038)	0.978	-	-
Serum chloride	1.006 (0.970–1.043)	0.746	-	-
serum calcium	0.246 (0.073–0.835)	**0.024**	-	-
Surgical management				
Root repalcement, n, (%)	1.084 (0.471–5.052)	0.474	-	-
Additional CABG, n, (%)	1.084 (0.221–5.323)	0.921	-	-
Operation time	1.001 (0.998–1.004)	0.654	-	-
CPB time	1.001 (0.997–1.004)	0.744	-	-
Perioperative calcium infusion	1.001 (0.996–1.006)	0.747	-	-
Lactic acid_max_	1.096 (1.033–1.163)	**0.002**	1.093 (1.022–1.169)	**0.010**
VIS_max_	1.024 (0.999–1.051)	0.062	1.005 (0.979–1.031)	0.712
Serum calcium				
High group	0.918 (0.485–1.739)	0.794	0.818 (0.403–1.661)	0.827
Medium group	Ref		Ref	
Continuous low group	2.981 (1.466–6.063)	**0.003**	2.454 (1.074–5.604)	**0.033**
Low serum calcium duration				
< 24 h	Ref		Ref	
24–48 h	1.481 (0.740–2.965)	0.267	1.534 (0.715–3.291)	0.272
> 48 h	2.629 (1.302–5.310)	**0.007**	3.595 (1.597–8.093)	**0.002**
ICU stay time	1.003 (0.979–1.028)	0.700	-	-
Hospital stays	1.003 (0.992–1.015)	0.557	-	-

*BMI* body mass index, *LVEF* left ventricular ejection fraction, *HB* hemoglobin, *CRP* C-reactive protein,Cr,creatinine, *BNP* B-type natriuretic peptide, *CABG* coronary artery bypass graft, *CBP* cardiopulmonary bypass, *VIS* vasoactive inotropic score

### Distinguish ability of the model

The ROC curve was plotted according to the prediction model, and the results showed an AUC of 0.909 (95% CI: 0.688–0.806, *P* < 0.001), a sensitivity of 77.5% and a specificity of 88%, suggesting that the model has a good discrimination. The ROC curve of prediction model is shown in Supplement 2A.

### Calibration degree of the model

The results of the H–L test showed χ2 = 9.106, *P* = 0.333 > 0.05, and the calibration curve was well fitted as shown by bootstrap method with 1000 repetitions (Supplement 2B), indicating that our predicted and observed values are close and in good agreement.

### Subgroup analysis

**Fig. 3:**
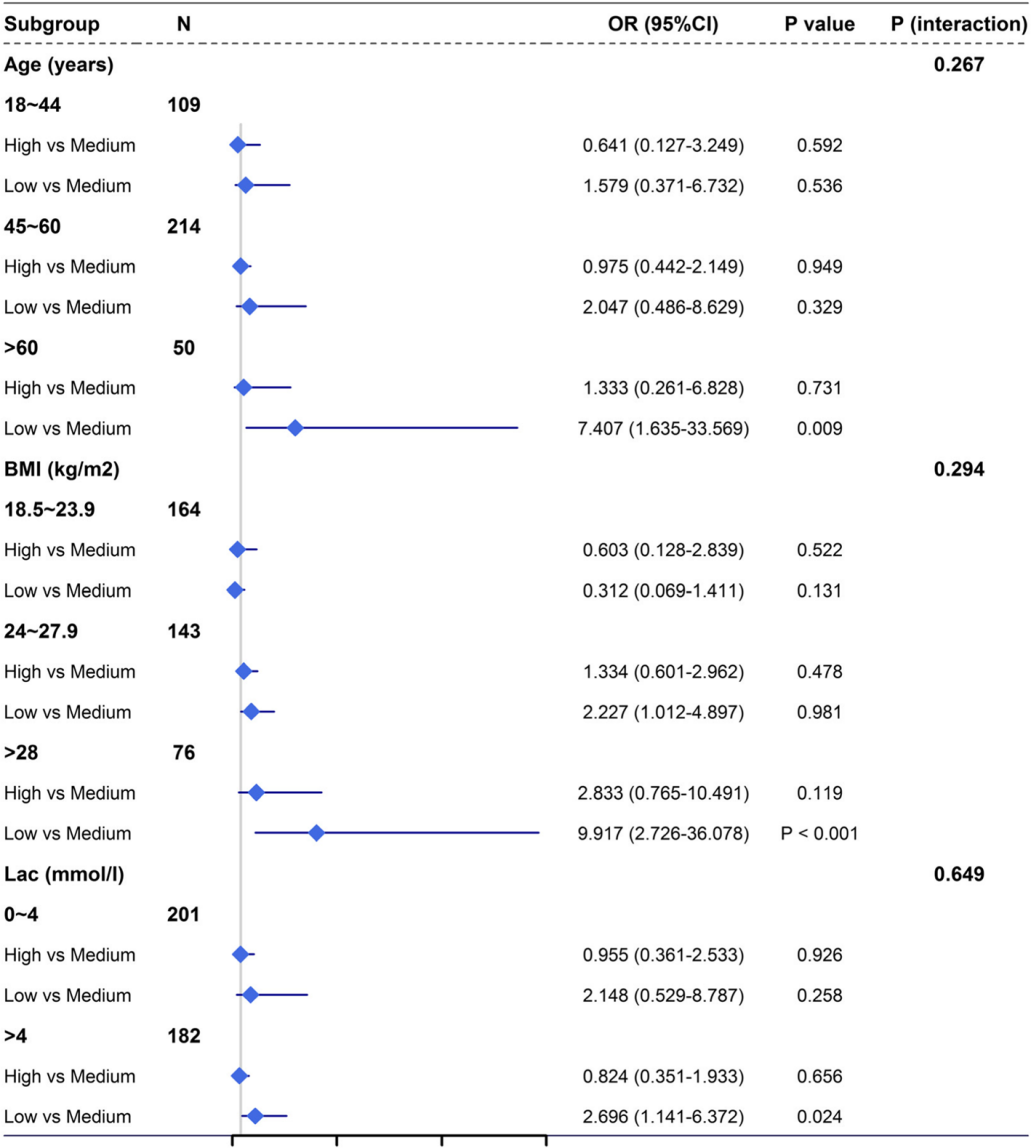
Subgroup analysis of poor prognosis and interaction of three groups

Consider that the effect of serum calcium on poor prognosis may be different between different populations. Age, BMI, and lactic acid levels were analyzed in the subgroups; of those ≥ 60 years, BMI > 28, and lactic acid > 4 mmol/L, the risk of poor prognosis in the persistent low-level group was significantly higher (*P* < 0.05) (P-interaction > 0.05) (Fig. 3).

## Discussion

To our knowledge, this study was the first to reveal distinct serum calcium trajectories among patients with AAAD by LCGM; and determine factors at baseline that predict these trajectories; and verify whether early changes in serum calcium were associated with prognosis outcomes. In addition, explore the prognosis of patients with AAAD and to analyze the occurrence of adverse prognosis and its risk factors. The results showed that: 1) among the 383 patients included, 22.2% were in the "high-level group", 67.6% in the "medium-level group", and 10.2% in the "continuous low-level group". 2) Compared to the medium-level group, patients in the continuous low-level group had more severe cardiovascular and cerebrovascular events, PMV, postoperative AKI, postoperative new arrhythmias, and mortality. 3) After adjustment for age, BMI, hypertension, LVEF, D-dimer, and arterial blood lactate, continuously low levels of serum calcium and low calcium for more than 48 h were independent risk factors for poor prognosis in patients with AAAD.

Three potential trajectories were identified through the growth mixed model: the high-level, medium-level, and continuous low-level groups. The proportion of patients in the medium level group (67.6%) was the highest. However, with increased intensity of treatment, serum calcium decreased significantly 24–72 h after surgery, resulting in the continuous low-level group accounting for 10.2% of patients. To our knowledge, there are no other studies on serum calcium changes in patients with AAAD. Polderman et al. [9] investigated the electrolyte level of patients after cardiac surgery and found that 2.9% of patients had a continuous low calcium level. However, Vianello et al. [12] found that serum calcium and magnesium levels were low in 54.5% of elderly patients with AAAD within 24 h after admission. The difference between the results of the above study and those of the present study may be related to the inclusion criteria and the methodology. The patients in our study had AAADs. The significant decrease in serum calcium homeostasis between 24 and 72 h may be due to hemodilution after CPB, diuretics, early fasting after surgery, gastrointestinal functional impairment, or neurohormonal activation. Based on our findings, nurses should assess patients postoperatively for serum calcium changes by increasing laboratory monitoring 24–72 h after surgery to promptly detect and correct low serum calcium levels and prevent clinical complications.

Logistic univariate and multivariate analyses showed that age, BMI, hypertension, lactic acid levels, and continuous low calcium levels for more than 48 h were risk factors for poor prognosis in patients with AAAD. The prognosis of patients aged ≥ 60 years (13.1%) was worse than that of the other demographic subgroups. Research has shown that patients who are elderly have various diseases/comorbidities [13]. Furthermore, older adult patients have reduced resistance and compensatory functions. This study showed that obesity was a risk factor for poor prognosis in patients with AAAD (*P* < 0.05). This result is consistent with that of Li et al. [14], who studied patients with acute thoracic aortic dissection who were obese. Particularly, patients who were morbidly obese had a high risk ratio of postoperative pulmonary complications and PMV. Conversely, a meta-analysis showed no difference in adverse outcomes between patients of normal weight and patients who were overweight or obese [15]. Although the difference was not significant, the poor prognosis of patients with abdominal obesity was higher than that of other patients, suggesting that different types of obesity may affect patient prognosis. This concept requires further clinical confirmation. The multivariate regression analysis in our study showed that a history of hypertension was associated with poor prognosis, which is consistent with other studies. Most patients with AAAD have myocardial hypertrophy, and the aortic valve is frequently involved in dissection, resulting in aortic insufficiency [16]. Simultaneously, a myocardium with severe coronary artery stenosis or obstruction and aortic valve insufficiency cannot be perfused adequately, resulting in myocardial ischemia and inadequate myocardial contractility during cardiac resynchronization. This may explain the poor prognosis. This study also found that elevated lactate levels were an independent risk factor for poor prognosis. Similar studies also showed that higher arterial blood lactate levels suggest poor prognosis [17]. When large vessels are affected, the corresponding tissues and organs have varying degrees of ischemia and hypoxia, increasing the lactic acid levels. Aortic dissection often requires cardiopulmonary CBP, increasing the probability of serious complications such as those affecting the nervous or renal systems.

Electrolyte disorders are associated with poor prognosis after cardiac surgery with difficulties such as increased postoperative complications, mortality, and prolonged ICU stay [18]. Electrolyte disorder is an internal environmental disorder and an important risk factor for multiple organ dysfunction [19]. Low serum calcium is a risk factor for poor prognosis in patients with AAAD, and low calcium levels for more than 48 h are more likely to cause poor prognosis. Although the underlying mechanism remains unclear, we speculate the following: first, serum calcium participates in the regulation of myocardial cells and vascular endothelial relaxation and contraction, and maintaining normal fluctuations can help stabilize cardiovascular physiological function. Studies have shown that low calcium intake increases the risk of hypertension. Low blood calcium levels may affect blood pressure by affecting vascular endothelial function, leading to abnormal blood pressure regulation mechanisms. Moreover, blood pressure fluctuates greatly, leading to disease deterioration and increased death risk [20]. Second, calcium ions are important in the coagulation process which involves tissue factor activation and platelet adhesion. In our study, blood coagulation time was significantly prolonged when serum calcium ions decreased, suggesting that continuous hypocalcemia may affect blood coagulation. The hematoma volume increases at admission, aggravates organ dysfunction, and increases the risk of postoperative bleeding. Third, serum calcium levels affect the stability of the blood–brain barrier. A previous study [21] showed that hypocalcemia can lead to interruption of cell adhesion in the barrier. A continuous decrease in the concentration of extracellular calcium ions leads to an increase in nerve cell apoptosis. Patient prognosis worsens with greater damage to neural function. Patients such as those in this study who underwent CBP are prone to electrolyte disorders resulting from surgical trauma and long duration of hypocalcemia.

The results of this study indicated that low serum calcium levels are also closely associated with arrhythmia, PMV, and AKI; the lower the level, the greater the incidence of arrhythmia, PMV, and AKI. According to previous studies, high serum calcium levels can prevent atrial fibrillation in patients after cardiac surgery [22], regulate electrolyte fluctuation within a relatively safe range, and reduce hemodynamic effects and arrhythmia incidence. Studies have shown that decreasing serum calcium ions interferes with myocardial function, leading to serious cardiovascular complications and organ dysfunction [23]. This study showed that patients with lower serum calcium levels are more likely to have a lower LVEF, which is related to PMV. Thus, patients with lower serum calcium levels were more likely to require PMV. Bi et al. [24] conducted a logistic multiple regression analysis of the risk factors of AKI in patients in the ICU after cardiac surgery, showing that hypocalcemia during an ICU stay is a high-risk factor for AKI, consistent with the results of the current study. When hemodynamic instability occurs, renal perfusion decreases. Hypocalcemia also triggers the opening of calcium channels in renal cells, resulting in calcium influx and overload, exacerbating kidney injury [25]. This suggests that clinicians should actively ascertain the causes of persistently low serum calcium levels in the early stages of patient care and promptly take targeted measures to prevent organ dysfunction.

This study had limitations. First, this was a single-center retrospective study, and multicenter prospective studies are needed to verify results. Second, we analyzed the change rule of serum calcium at admission and within 72 h after surgery. Further studies with a longer follow-up period are required to comprehensively investigate the changes in patient electrolyte levels perioperatively. Finally, this study only included patients with AAAD. The effect of serum calcium on the prognosis of other patients undergoing cardiac surgery remains to be elucidated.

## Conclusion

In conclusion, this study showed that continuous low serum calcium levels can increase the risk of poor prognosis in patients with AAAD after surgery. Further prospective studies are required to determine the optimal serum calcium levels for patients with AAAD.

### Supplementary Information

**Additional file 1: Supplement 1.** Nomogram to estimate risk of poor prognosis of patients with acute type A aortic dissection. **Supplement 2.** (A)Receiver operator characteristic curve (B)Calibration curve of nomogram mode. **Supplement 3.** Clinical data in three groups stratified by serum calcium trajectories after PSM. **Supplement 4.** Poor prognosis in three groups stratified by serum calcium trajectories after PSM.

## Data Availability

The datasets generated and/or analysed during the current study are not publicly available due to individual privacy, but are available from the corresponding author on reasonable request.

## Abbreviations

AAD: Acute aortic dissection; CTA: Computed tomography angiography
IQR: Interquartile range
