# Supplementary material for: Analysis of serum calcium change trajectories and prognostic factors in patients with acute type A aortic dissection
Source: BMC Surg. 2023 Nov 27;23:362. doi: 10.1186/s12893-023-02249-3 (PMC10683301; doi:10.1186/s12893-023-02249-3)
Supplement: Supplementary file 1 — Additional file 1: Supplement 1. Nomogram to estimate risk of poor prognosis of patients with acute type A aortic dissection. Supplement 2. (A)Receiver operator characteristic curve (B)Calibration curve of nomogram mode. Supplement 3. Clinical data in three groups stratified by serum calcium trajectories after PSM. Supplement 4. Poor prognosis in three groups stratified by serum calcium trajectories after PSM. [file 12893_2023_2249_MOESM1_ESM.docx]

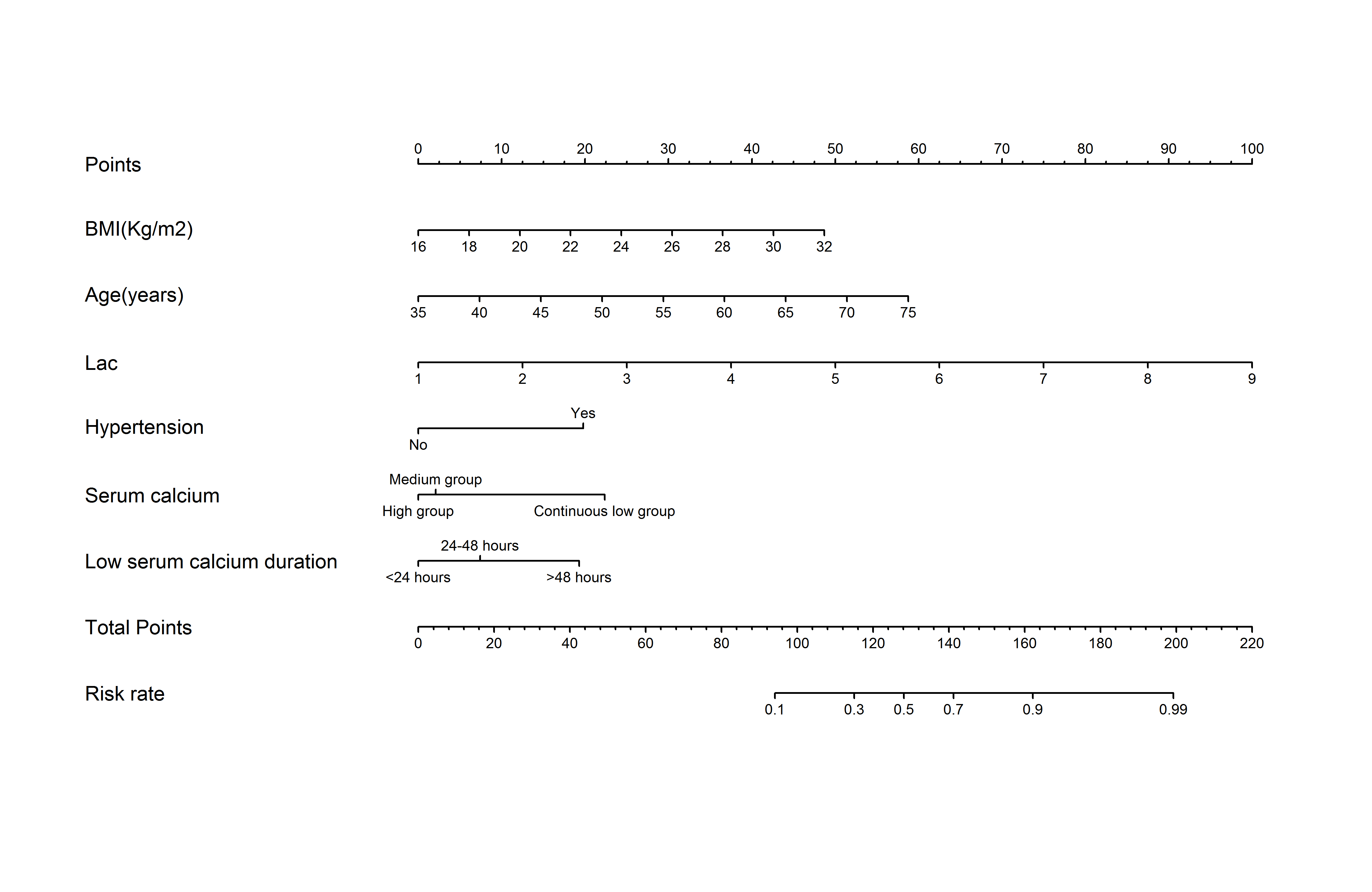


**Supplement 1 .** Nomogram to estimate risk of poor prognosis of patients with acute type A aortic dissection

A


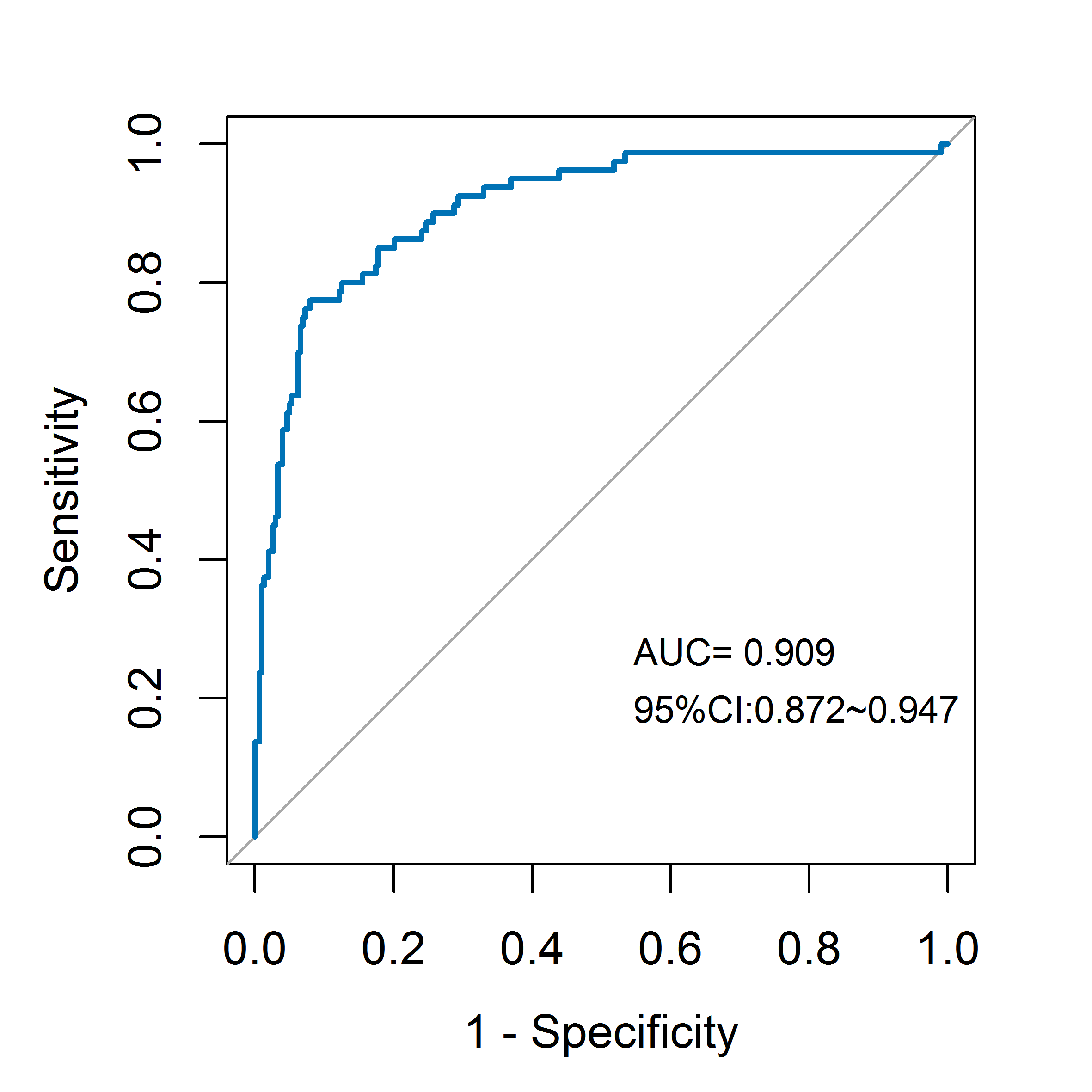


B


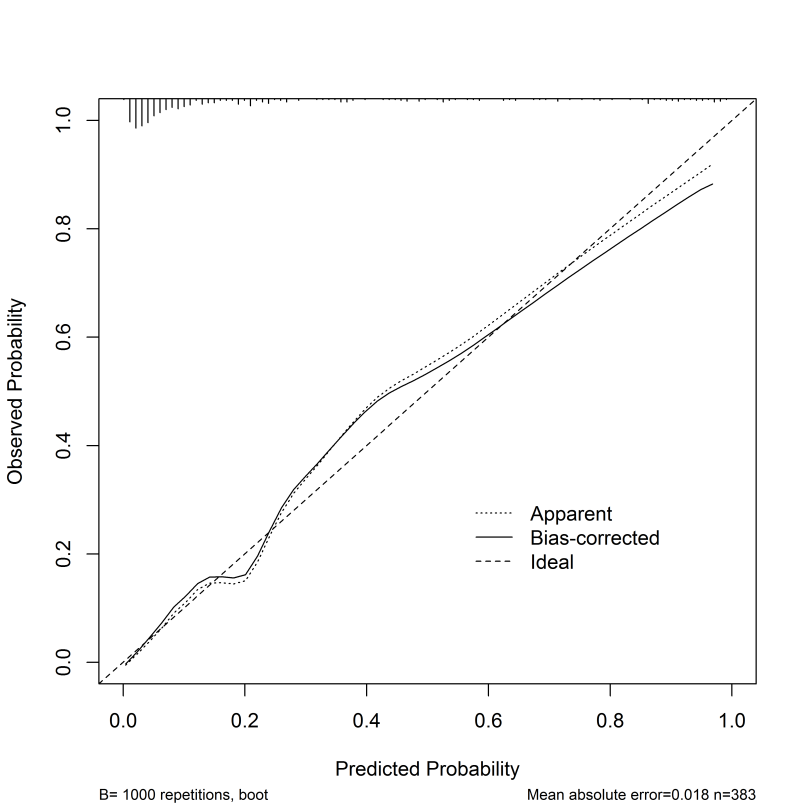


**Supplement 2 .** (A)Receiver operator characteristic curve (B)Calibration curve of nomogram mode.

**Supplement 3 .** Clinical data in three groups stratified by serum calcium trajectories after PSM

| Variables | Total  (n=99) | High group  (n=33) | Medium group  (n=33) | Continuous low group  (n=33) | *P* -value |
| --- | --- | --- | --- | --- | --- |
| Age(years), mean (SD) | 50.2±7.3 | 50.2±7.6 | 50.1±8.6 | 49.3±8.5 | 0.606 |
| Female, n, (%) | 16(16.2) | 5(15.2) | 3(9.1) | 7(21.2) | 0.390 |
| BMI (kg/m^2^), mean (SD) | 25.8±3.3 | 25.7±2.9 | 25.8±2.9 | 26.1±3.3 | 0.991 |
| Smoking, n, (%) | 49(49.5) | 20(60.6) | 17(51.5) | 12(36.4) | 0.138 |
| Drinking, n, (%) | 36(36.4) | 9(27.3) | 17(51.5) | 10(30.3) | 0.083 |
| Medical history |  |  |  |  |  |
| Diabetes mellitus, n, (%) | 9(9.1) | 4(12.1) | 2(6.1) | 3(9.1) | 0.693 |
| Hypertension, n, (%) | 82(82.8) | 26(78.8) | 28(84.8) | 28(84.8) | 0.753 |
| Prior resuscitation, n, (%) |  |  |  |  |  |
| ≥ 3 organs, n, (%) | 4(4.0) | 1(3.0) | 2(6.1) | 1(3.0) | 0.771 |
| Echocardiography results |  |  |  |  |  |
| LVEF (%), mean (SD) | 62.28±7.83 | 63.88±5.94 | 61.35±9.61 | 61.67±7.53 | 0.391 |
| Laboratory results |  |  |  |  |  |
| Hb(g/L), mean (SD) | 123.80±15.87 | 125.09±14.21 | 123.75±13.63 | 122.57±19.50 | 0.816 |
| CRP (mmol/L), median (IQR) | 12.9(4.7, 31.7) | 12.0(4.6, 31.3) | 12.7(4.6, 33.7) | 12.6(4.6, 31.3) | 0.110^a^ |
| Cr(mmol/L), median (IQR) | 92(69, 128) | 103(71, 141) | 82(67, 123) | 90(68, 129) | 0.328^a^ |
| BNP (mmol/L), median (IQR) | 328(120, 901) | 326(125, 898) | 326(123, 900) | 367(126, 920) | 0.799^a^ |
| Serum albumin(g/l), median (IQR) | 43(48,49) | 44(42,46) | 45(41,47) | 43(40,48) | 0.612^a^ |
| Serum potassium(mmol/l), mean (SD) | 4.11±0.54 | 4.00±0.51 | 3.98±0.53 | 4.07±0.58 | 0.489 |
| Serum sodium(mmol/l), mean (SD) | 135.39±5.84 | 135.28±5.39 | 135.51±4.00 | 135.76±4.23 | 0.715 |
| Serum chloride(mmol/l), mean (SD) | 98.14±6.21 | 98.82±4.80 | 98.33±5.10 | 95.39±5.09 | 0.347 |
| Lactic acid_max_ (mmol/l), median (IQR) | 3.7(2.3, 6.6) | 4.1(2.3, 8.0) | 3.9(2.6, 6.9) | 4.2(2.8, 7.0) | 0.133^a^ |

^a^Kruskal-Wallis H; ^b^Fisher's exact test

BMI,body mass index; LVEF,left ventricular ejection fraction; HB,hemoglobin; CRP,C-reactive protein; Cr,creatinine; BNP,B-type natriuretic peptide; PSM, propensity score matching.

**Supplement 4 .** Poor prognosis in three groups stratified by serum calcium trajectories after PSM

| Outcomes | Total  (n=99) | High group  (n=33) | Medium group  (n=33) | Continuous low group  (n=33) | *P* -value |
| --- | --- | --- | --- | --- | --- |
| ICU stay time(day), median (IQR) | 6(4, 10) | 7(3, 9) | 6(3, 9) | 6(4, 9) | 0.772^a^ |
| Hospital stays(day), median (IQR) | 22(16, 31) | 20(16, 28) | 25(19, 34) | 25(16, 30) | 0.077^a^ |
| Death in hospital, n, (%) | 17(17.2) | 3(9.1) | 4(12.1) | 10(30.3) | **0.047** |
| Poor prognosis, n, (%) | 30(30.3) | 6(18.2) | 9(27.3) | 15(45.5) | **0.049** |

^a^Kruskal-Wallis H
